# iRDA: a new filter towards predictive, stable, and enriched candidate genes

**DOI:** 10.1186/s12864-015-2129-5

**Published:** 2015-12-09

**Authors:** Hung-Ming Lai, Andreas A. Albrecht, Kathleen K. Steinhöfel

**Affiliations:** Algorithms and Bioinformatics Research Group, Department of Informatics, King’s College London, Strand, London, WC2R 2LS UK; School of Science and Technology, Middlesex University, Burroughs, London, NW4 4BT UK

**Keywords:** Cancer phenotype prediction, Feature selection and classification, Microarray, Prognosis gene signature, Transcriptomic profiling

## Abstract

**Background:**

Gene expression profiling using high-throughput screening (HTS) technologies allows clinical researchers to find prognosis gene signatures that could better discriminate between different phenotypes and serve as potential biological markers in disease diagnoses. In recent years, many feature selection methods have been devised for finding such discriminative genes, and more recently information theoretic filters have also been introduced for capturing feature-to-class relevance and feature-to-feature correlations in microarray-based classification.

**Methods:**

In this paper, we present and fully formulate a new multivariate filter, iRDA, for the discovery of HTS gene-expression candidate genes. The filter constitutes a four-step framework and includes feature relevance, feature redundancy, and feature interdependence in the context of feature-pairs. The method is based upon approximate Markov blankets, information theory, several heuristic search strategies with forward, backward and insertion phases, and the method is aiming at higher order gene interactions.

**Results:**

To show the strengths of iRDA, three performance measures, two evaluation schemes, two stability index sets, and the gene set enrichment analysis (GSEA) are all employed in our experimental studies. Its effectiveness has been validated by using seven well-known cancer gene-expression benchmarks and four other disease experiments, including a comparison to three popular information theoretic filters. In terms of classification performance, candidate genes selected by iRDA perform better than the sets discovered by the other three filters. Two stability measures indicate that iRDA is the most robust with the least variance. GSEA shows that iRDA produces more statistically enriched gene sets on five out of the six benchmark datasets.

**Conclusions:**

Through the classification performance, the stability performance, and the enrichment analysis, iRDA is a promising filter to find predictive, stable, and enriched gene-expression candidate genes.

**Electronic supplementary material:**

The online version of this article (doi:10.1186/s12864-015-2129-5) contains supplementary material, which is available to authorized users.

## Background

Prognosis gene signatures for the discovery of biological markers in carcinogenesis studies and the diagnosis of diseases is one of the essential areas in biomedical research. High-throughput screening technologies (HTS), such as microarrays, are able to examine more than a hundred thousand of oligonucleotide probes in parallel, which allows the interrogation of thousands of mRNA transcripts in a single experiment. To date, transcriptome analysis using HTS gene expression profiling has become a useful approach that can provide a stronger predictive power of clinical changes than the diagnostic testing procedures used in pathology [1, 2]. Out of thousands of interrogated transcripts in the cell of interest, a small subset of genes is assumed to be differentially expressed and is subject to change [3]. The exploration of differentially expressed genes that contribute to a better prediction can be referred to as feature selection.

Feature selection is a technique of reducing the feature dimension of sample instances, where a subset of features is selected without creating new features from the original form of the features. This technique is widely used in data mining, machine learning and pattern recognition, and has also been applied to the field of bioinformatics [4]. Known to be an NP-complete problem [5], feature subset selection not only finds a subset of relevant features for the use of a model construction but also looks into the minimal subset that optimises the best predictive model. This is actually based on the principle of parsimony [6], i.e., seeking a model that has as few as possible variables to fit the data sufficiently. Gene expression microarray experiments are often affected by noise that is caused by the experimental design of the underlying microarray technique, the stages of sample preparation, and the hybridisation processes of oligonucleotide probes [7]. Several statistical and computational methods have been introduced to cope with the probe level data in recent years [8–11]. Besides the unavoidable technical noise, a typical scenario in the context of discovering gene-expression candidate genes is that there are many thousands of genes to be interrogated, but only tens to a hundred of clinical samples are available [12]. The curse of dimensionality makes the process of selecting relevant genes even more challenging.

Filter, wrapper, and embedded methods are the three main types of feature selection techniques, where the taxonomy is based on the degree of interaction within a classification method [4]. A filter, being either univariate or multivariate, does not use a classifier within its selection scheme and takes only the intrinsic characteristics of sample instances into account in order to quantify the association between features and phenotypes. SAM [13] and LIMMA [14] are two examples of univariate filters in the domain of individual selections of differentially expressed genes, based on random permutations (nonparametric) and *t*-statistics (parametric), respectively. On the other hand, a multivariate filter, such as CFS [15, 16], considers feature interactions and therefore does not evaluate features independently, which is sometimes referred to as space search methods [17]. A wrapper (deterministic or randomised) measures the predictive power of a feature subset by using a classification model which a repetitive selection scheme is wrapped around [18, 19]. Due to small sample sizes and an abundance of features, a wrapper is usually prone to overfitting and computationally expensive in spite of the benefit of its multivariate nature. While sequential search (backward, forward, floating, or best-first) is deterministic [20], simulated annealing and genetic algorithms can be regarded as classical randomised search methods [21–23]. Search procedures embedded into a given learning algorithm where features are ranked or weighted in the context of a classification task are called embedded methods. Popular embedded methods are SVM-RFE-like [24–27] and Random-Forest [28, 29]. Both methods interact well with classifiers, are of multivariate nature, and require less computational time compared to a wrapper. Nowadays robustness or stability of feature selection are one of the major issues. Several techniques [30–32] have been devised for making feature selection more stable for biomarker reproducibility. Of particular interest is the ensemble approach [30]. The approach uses a sampling technique to generate numerous different selectors and combines the components into a consensus ranking list. With regard to the stability of feature selection, we refer the reader to [33–35].

Recently, feature selection methods using information theory have been devised for feature-to-class relevance and feature-to-feature correlations, including a probabilistic interpretation based upon the conditional likelihood maximisation in order to unify information theoretic feature selection [36]. We consider in more detail three information theory-based multivariate filters that exemplify an approximation of higher order gene interactions and aim at the selection of a gene subset. The methods are compared to a new gene-expression candidate gene filter proposed in the present paper. The first method is called the minimum-Redundancy and Maximum-Relevance framework (mRMR). It uses mutual information to manage the tradeoff between the deduction from redundant features and the gain from relevant features [37]. The Conditional Mutual Information Maximization (CMIM) method utilises the so-called minimum operator of conditional mutual information for the evaluation of relevant features that are conditioned on the selected feature subset by using only pairwise feature statistics [38]. Whereas mRMR and CMIM introduce evaluation criteria, the Fast Correlation-Based Filter (FCBF) uses symmetrical uncertainty and designs an efficient backward elimination scheme for the removal of irrelevant and redundant features [39]. The three filters all consider feature relevance and feature redundancy, but they still neglect feature interdependence in favour of moderate computational complexity. Despite the lesser relevance of neglected features, they could, however, exhibit a strong discrimination when combined with other features and might reveal interactions within a set of candidate genes.

In this paper, we present and fully formulate a new multivariate filter, iRDA, designed for the exploration of cancer-related candidate genes under a HTS gene expression profiling experiment. The filter is based on information theory, approximate Markov blankets, and several heuristic search strategies. Being a four-step framework, iRDA takes into account a number of feature properties that include feature relevance, feature redundancy, and feature interdependence in the context of feature-pairs. The iRDA filter is a data-driven approach that does not employ *a priori* biological information and the filter can properly tackle interdependent features through the subtle design of the underlying algorithmic procedures. Additionally, the filter produces a small number of discriminative genes for improved phenotype prediction, which is advantageous for the domain user since a small number of candidate genes supports greater efficiency of *in vitro* validation. To demonstrate the strengths of iRDA, three performance measures, two evaluation schemes, two sets of stability measures, and the gene set enrichment analysis (GSEA) have all been used in our experiments. Its effectiveness has been validated by using eleven gene expression profiling data (seven well-known cancer benchmarks and four different disease experiments). The experimental results show that iRDA is stable and able to discover gene-expression candidate genes that are statistically significant enriched and constitute high-level predictive models.

## Preliminaries

### Domain description

**Fig. 1:**
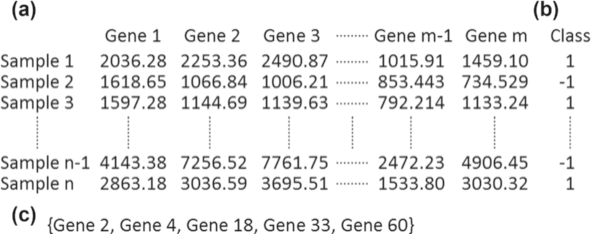
Cancer classification using high-throughput screening technologies. **a** An example of gene expression profiling data. The experimental dataset contains *n* samples and each sample has *m* interrogated genes (*m*≥*n*). **b** The extracted samples of an experiment are labeled according to their phenotypes or different types of cell lines. Both a gene expression matrix and a class vector form the input of gene selection for cancer classification. **c** A subset *G* of significant genes is obtained as an output for a certain cancer classification, which is a so-called gene signature

In this section, the domain of HTS gene selection for phenotype prediction is briefly described. Given a gene expression dataset $D=\{\mathrm {X}\in \mathbb {R}^{m},Y\in \mathbb {R}\}={\{(x_{i},y_{i})\}}^{n}_{i=1}$, where *D* consists of *n* samples X labeled by a class vector *Y* (Fig. 1b), and each sample is profiled over *m* gene expressions, i.e. $x_{i}={\{(x_{i1},\cdots,x_{\textit {im}})\}}^{n}_{i=1},m\gg n$ (Fig. 1a). The task is to find a small number of discriminating genes (from tens to a hundred) (Fig. 1c) for clinical classification to be validated experimentally and to identify a gene signature for a specific disease. To address the issue of HTS-based gene signatures, one can refer to the task as a feature selection problem. Let *F* be a full set of features (genes) $F={\{f_{i}\}}^{m}_{i=1}$, then feature selection aims at choosing a feature subset *G*⊂*F* that maximizes the prediction performance; moreover, if one tries to minimise *G*, a parsimonious subset is sought for.

### Information theory basics

Entropy is the rationale behind information theory and is an intuitive measure to evaluate the uncertainty of a random variable. Given a variable, the entropy is computed at the level of probability distributions [40]. Let X be a nominal random variable, then the Shannon *entropy* is defined as

(1) $$ \text{H(X)}=-{\sum}_{x\in X} ~p(x)~{\log}~ p(x),  $$

where the *x* denote the values of the random variable X, and *p*(*x*) is the marginal probability distribution of X. Unlike conventional statistics, an entropy-based measure does not make any *a priori* assumptions. This differs, for instance, from the student’s t-test, where the values have to be normally distributed. Further information quantities can be defined through applying probability theory to the notion of entropy. The *conditionalentropy* of X given Y is represented as

(2) $$ \text{H(X|Y)}=-{\sum}\,p(y)~{\sum}\,p(x|y)~ {\log}~ p(x|y),  $$

where *p*(*x*|*y*) is the conditional probability of X given the observed values of Y. This quantity evaluates how much uncertainty of X is left given that the value of another random variable Y is known. Similarly, the *jointentropy* of two random variables X and Y is denoted by

(3) $$ \text{H}(\text{X,Y})=-{\sum}~{\sum}~p(\text{\textit{x,y}})~{\log}~p(\text{\textit{x,y}}),  $$

where *p*(*x,y*) is the joint probability distribution of X and Y. It quantifies the amount of information needed to describe the outcome of two jointly distributed random variables. Another important information theoretic measure, *mutual information*, quantifies the amount of information shared by two random variables X and Y. The quantity can be defined according to

(4) $$ \text{MI}(\text{X,Y})=\text{H(X)}-\text{H(X|Y)}.  $$

The measure is symmetric and non-negative, and if the value equals zero, then the two variables are statistically independent. The mutual information of X and Y can also be conditioned on a variable Z as *conditional mutual information*, which is defined by

(5) $$ \text{CMI}(\text{X,Y|Z})=\text{H(X|Z)}-\text{H(X|Y,Z)}.  $$

The quantity measures the information amount shared between X and Y, if Z is known. Finally, we introduce *symmetrical uncertainty*, a measure that will be heavily utilized in our gene selection framework throughout the paper. The measure can be viewed as one type of normalised mutual information and is defined as

(6) $$ \text{SU}_{\text{X,Y}}=2\left[\frac{\text{H(X)}-\text{H(X|Y)}}{\text{H(X)}+\text{H(Y)}}\right].  $$

Similar to the joint entropy, the joint symmetrical uncertainty can be defined as

(7) $$ \text{SU}_{\mathrm{X}_{1},\mathrm{X}_{2};\mathrm{Y}}=2\left[\frac{\mathrm{H}(\mathrm{X}_{1},\mathrm{X}_{2}) -\mathrm{H}(\mathrm{X}_{1},\mathrm{X}_{2}|\mathrm{Y})}{\mathrm{H}(\mathrm{X}_{1},\mathrm{X}_{2})+\text{H(Y)}}\right].  $$

### Feature relevance

Feature subset selection is to find a subset of the original features of a dataset such that a classifier generates the highest accuracy of classification upon the reduced data that only contains the selected features. Kohavi and John (hereafter KJ) [41] addressed the issue of finding a good feature subset and its relation to which features shall be included by partitioning features into three types of strong relevance, weak relevance, and irrelevance. Given a class variable *C*, a set of features *F*, a feature *f*_*i*_∈*F*, and *F*_*i*_=*F*∖*f*_*i*_, the KJ feature types are defined by the conditional probability below.

#### **Definition1**.

KJ-Strong Relevance.A feature variable *f*_*i*_ is strongly relevant iff there exists an assignment of values $\tilde {c}, \widetilde {f_{i}}, \widetilde {F_{i}}$ for which

(8) $$ p\left(C=\tilde{c}|\,f_{i}=\widetilde{f_{i}},F_{i}=\widetilde{F_{i}}\right)\ne p\left(C=\tilde{c}|F_{i}=\widetilde{f_{i}}\right)  $$

or

(9) $$ p(C|\,f_{i},F_{i}) \ne p(C|F_{i}) \; \text{for short.}  $$

#### **Definition2**.

KJ-Weak Relevance.A feature *f*_*i*_ is weakly relevant iff

(10) $$ \begin{aligned} p\left(C|\,f_{i},F_{i}\right)=p\left(C|F_{i}\right) \; \text{and} \; \exists F_{i}^{\prime}\subset F_{i}\\ \; \text{such that} \; p\left(C|\,f_{i},F_{i}^{\prime}\right)\ne p\left(C|F_{i}^{\prime}\right). \end{aligned}  $$

#### **Definition3**.

KJ-Irrelevance.A feature *f*_*i*_ is irrelevant iff

(11) $$ \forall F_{i}^{\prime}\subseteq F_{i}, p\left(C|\,f_{i},F_{i}^{\prime}\right)=p\left(C|F_{i}^{\prime}\right).  $$

Kohavi and John used the above theoretical representations to claim that two degrees of feature relevance (strong and weak) are required in terms of an optimal classifier. The removal of a strongly relevant feature will result in performance deterioration of the classifier. For a weakly relevant feature *f*_*i*_, there exists a subset of features, $F_{i}^{\prime }$, such that the performance of the classifier running on $F_{i}^{\prime }$ is worse than the performance on $F_{i}^{\prime }$ with the inclusion of *f*_*i*_. The loss of discriminative power is reflected by the symbol ≠ in the KJ representation [41]. In short, the strongly relevant feature is indispensable in the KJ sense and cannot be removed without loss of prediction accuracy, while a weakly relevant feature can sometimes contribute to classification performance.

Similar to KJ definitions, we can define a strongly relevant feature-pair *f*_*ij*_ given two jointly distributed random variables *f*_*i*_ and *f*_*j*_ (or *f*_*ij*_).

#### **Definition4**.

KJ-Strongly Relevant Feature-pair.

A feature-pair *f*_*ij*_ is strongly relevant iff

(12) $$ p\left(C|\,f_{ij},F_{ij}\right)\ne p\left(C|F_{ij}\right).  $$

where *F*_*ij*_ denotes the feature set *F* excluding *f*_*i*_ and *f*_*j*_ at the same time.

A feature-pair is referred to as a united-individual and must be selected together during the process of selection. The strong relevance of a feature-pair will be the basis for the framework presented in our paper for finding HTS gene-expression candidate genes.

### KJ-relevance, correlation, and discretization

Kohavi and John proposed two families of feature relevance (strong and weak) and claimed that a classifier should be taken into account when selecting relevant features. Therefore, Kohavi and John used a wrapper approach to investigate feature relevance by an optimal classifier in practical selection scenarios, such that the prediction accuracy of the classifier was estimated using an accuracy estimation technique [41]. On the other hand, correlation is widely used in filter-based feature selection for relevance analysis [15, 39] with the use of a correlation measure. A correlation-based filter employs the following assumption: if a feature variable (*f*_*i*_) is highly correlated with a class variable (*C*), then the case of strong relevance is expected [15]. A higher correlation value implies a stronger feature relevance.

There are various measures for the correlation between two random variables. A typical correlation measure is Relief - it assigns a relevant weight to each feature that represents the relevance of the feature variable to the class variable [42]; the measure has been used in CFS [15]. Other popular correlation measures are based on the notion of entropy in the context of feature selection filters [36], which is mainly used in this paper and requires the continuous gene expression data need to be discretized for the calculation of entropy-based quantities. Here, we discretize continuous features using the scheme presented in [36, 37]. Given the mean (*μ*) and standard deviation (*σ*) of expression data for a gene across all l, any values smaller than *μ*−*σ*/2 are substituted by 1; any values between *μ*−*σ*/2 and *μ*+*σ*/2 are replaced by 3; any values larger than *μ*+*σ*/2 are transformed to 5. Like other correlation-based filters, a measure to quantify the correlation between two random variables needs to be defined. In the present framework, this measure is called R-Correlation and we propose four types of R-Correlation, where each type applied to a different stage of our four-step selector of candidate genes.

#### **Definition5**.

R-Correlation.The four types of correlation are:

- (a) R1-Correlation expresses the correlation between the feature *f*_*i*_ and the class *C*, denoted by *R*(*f*_*i*_,*C*);
- (b) R2-Correlation expresses the correlation between the feature-pair *f*_*ij*_ and the class *C*, denoted by *R*(*f*_*ij*_,*C*);
- (c) R3-Correlation expresses the correlation between the feature *f*_*i*_ and the feature *f*_*j*_, denoted by *R*(*f*_*i*_,*f*_*j*_);
- (d) R4-Correlation expresses the correlation between the feature *f*_*i*_ and the class *C* given a seed feature set *M*_*s*_, denoted by *R*(*f*_*i*_,*C*|*M*_*s*_).

Here, *R*(X,Y) measures the degree of correlation between X and Y (Definition 5(a)-(c)), and *R*(X,Y|Z) quantifies their correlation conditioned on an additional variable Z (Definition 5(d)). Based on the generic definition, a number of suitable correlation measures - either linear or nonlinear - can therefore be applied to our framework. In the present paper, we choose the information-theoretic measures of Shannon *entropy* to calculate the four types of R-Correlation (based upon the above mentioned discretized data). The correlation measures are $\phantom {\dot {i}\!}\text {SU}_{\text {\textit {X,Y}}}, \text {SU}_{X_{1},X_{2};Y}$, and CMI (*X,Y*|*Z*); defined in Eqs. 6, 7, and 5. The details of how the correlations are calculated and where the four types of R-Correlation are applied are shown in Table 1.

**Table 1:** The calculation and use of four types of R-Correlation

R-Correlation	Calculation	Applied in/to
R1: *R*(*f*_*i*_,*C*)	SU_*i,C*_	$\{\Omega _{k}\},M_{s},G\! \subseteq \!\cup _{u}{M_{s}^{p}}[\!u]$
		*ε*-Estimation
R2: *R*(*f*_*ij*_,*C*)	SU_*i,j*;*C*_	Strongly Relevant *f*_*ij*_
		*ε*-Estimation
R3: *R*(*f*_*i*_,*f*_*j*_)	SU_*i,j*_	*ε*-Estimation
R4: *R*(*f*_*i*_,*C*|*M*_*s*_)	CMI (*f*_*i*_,*C*|*M*_*s*_)	Feature Redundancy

*R*(*f*_*i*_,*C*) (R1-Correlation) is used to establish the structure of “Relevance-based K-Partition” ({*Ω*_*k*_}), which is being introduced in Definition 9. We also use the R1-Correlation for arranging the order of features that form a seed feature set *M*_*s*_ (Definition 11) and to aggregate candidate genes *G* from a set of parsimonious sets ${M_{s}^{p}}[\!u]$ (Definition 14). The strength of *R*(*f*_*ij*_,*C*) (R2-Correlation) is utilised for exploring KJ-strongly relevant feature-pairs *f*_*ij*_; see Definition 6. To estimate the crucial threshold *ε* in Definition 15, R1-, R2-, and R3-Correlations (*R*(*f*_*i*_,*f*_*j*_)) are required. Finally, *R*(*f*_*i*_,*C*|*M*_*s*_) (R4-Correlation) is employed as a conditional independence test for identifying redundant features with respect to a subset of features (Corollary 1).

## Methods

### Notions and fundamental principles

We introduce a number of fundamental concepts that constitute a filter for high-throughput screening gene selection. In our previous study [43], we have found that feature-pairs would play more important roles than individual features in the context of discovering candidate genes for cancer classification via a “Ratio by Correlation” plot. By utilising a suitable correlation measure, in general, a feature-pair variable (*f*_*ij*_) can be highly correlated with a class variable *C* if compared to a single feature variable (*f*_*i*_) [43]. Also, feature-pairs having high correlation values are combinations of different types of features in the context of strong and weak correlations; that is, it could be a pair of strongly correlated features; a strongly correlated feature & a weakly correlated feature; or a pair of a weakly correlated features. Thus, while searching for strongly relevant feature-pairs, not only strongly relevant features can be selected, but also putative weakly relevant features can be included, i.e., features of weak relevance are sometimes able to contribute to the classification performance when combined with other features. Consequently, a feature-pair could have more potential than a single feature when dealing with feature interdependency that takes gene synergy into account. This leads to the following criteria for finding potential feature-pairs, assuming that the more likely a feature-pair *f*_*ij*_ correlates with a class variable *C*, the more likely it is KJ-strongly relevant.

#### **Definition 6**.

KJ-Strongly Relevant Feature-pairs.For a fixed threshold *ε*≥0, a feature-pair *f*_*ij*_ is considered to be (*ε*-)KJ-strongly relevant iff

(13) $$ \quad R\left(\,f_{ij},C\right) > \varepsilon.  $$

Within our framework, we apply the concept of a Markov blanket in order to be able to identify minimal subsets of discriminative features resulting from the exploration of KJ-strongly relevant feature-pairs by using the measures of R2-correlation. The concept of Markov blankets was introduced in [44] and was incorporated into optimal feature selection by Koller and Sahami [45], with the assumption that the Markov blanket MB of a target variable *C* is independent of any *f*_*i*_∈*F*∖MB; the FCBF method [39] extended the approach from [45] to efficiently remove redundant features, based on the search for an approximate Markov blanket. Additionally, Tsamardinos and Aliferis [46] considered the connection between KJ-relevance and the Markov blanket of a target variable in a Bayesian Network faithful to some data distribution, which aims at building the minimal subset of features according to the following definition:

#### **Definition 7**.

Markov Blanket. A Markov blanket, MB, is a minimal set of features such that ∀*f*_*i*_∈*F*∖MB,

(14) $$ \quad p(C|\,f_{i}),\text{MB})=p(C|\text{MB}).  $$

Tsamardinos and Aliferis [46] showed that the blanket is unique and that it also coincides with the case of KJ-strongly relevant features under the assumption of “faithfulness” (see Definition 13 in [46]), which can be summarised in the following theorem (see Theorem 5 in [46] along with the proof).

#### **Theorem1**.

In a faithful BN, a feature *f*_*i*_∈*F* is KJ-strongly relevant if and only if *f*_*i*_∈MB.

Since we focus on feature-pairs, we extend the notion of Markov blankets accordingly:

#### **Definition 8**.

Markov Blanket for Feature-pairs.A Markov blanket for feature pairs, M_fp_, is a minimal set of feature-pairs such that ∀*f*_*ij*_∈*F*∖M_fp_,

(15) $$ \quad p(C|\,f_{ij},\mathrm{M}_{\text{\scriptsize fp}})=p(C|\mathrm{M}_{\text{\scriptsize fp}}).  $$

#### **Assumption**.

*f*_*ij*_∈M_fp_ iff *f*_*ij*_ is KJ-strongly relevant.

Typically, there is a huge number of interrogated genes in high-throughput gene expression profiling. Therefore, finding an exact Markov blanket appears to be impractical. Similar to the strategy proposed in [39] regarding the FCBF method, we aim at finding an approximate Markov blanket for the problem of discriminative gene discovery. High-throughput gene expression profiling returns only a relatively small number of differentially expressed genes, and the correlation values between a feature variable and a class variable are exponentially distributed. Thus, we propose the K-partition of the feature (gene) space with regard to relevance, which is a key component of our framework.

#### **Definition 9**.

Relevance-based K-Partition.

Given a feature space *F* and ${\{\Omega _{k}\}}_{k=1}^{K}$, where ${\Omega }_{k}=\left \{{f_{i}^{k}}|i=1,\cdots,|{\Omega }_{k}|\right \}$. If

(16) $$ \begin{aligned} (a) \quad \forall 1 \le i<|\Omega_{k}|, R\left({f_{i}^{k}},C\right) \ge R\left(\,f_{i+1}^{k},C\right); \end{aligned}  $$

(17) $$ \begin{aligned} &(b) \quad \text{Let} \; \widetilde{\Omega}_{k} \; \text{be the mean of}\; R\left({\,f_{i}^{k}},C\right) \;\text{in} \; {\Omega}_{k},\\ &\qquad\qquad \text{then} \; \forall 1 \le i<K, \widetilde{\Omega}_{k}>\widetilde{\Omega}_{k+1}; \end{aligned}  $$

(18) $$ \begin{aligned} &(c) \quad {\Omega}_{k} \cap \Omega_{k+1}= \emptyset \;\text{and}\;\\ &\qquad F=\Omega_{1} \cup \Omega_{2} \cup \cdots \cup \Omega_{K}, \end{aligned}  $$

then ${\{\Omega _{k}\}}_{k=1}^{K}$ is called a relevance-based K-partition of *F*. Note that the symbol |⋯| represents the cardinality of a set.

The proposed partition orders features with regard to the relevance of a class variable within a partition and between partitions. Features in the same partition can be viewed as having a similar scale of relevance, while features from two remote partitions do belong to two distinct feature types. For example, if we assume that strongly/weakly relevant features are in *Ω*_1_,…,*Ω*_*K*−1_, then *Ω*_*K*_ can be regarded as the collection of irrelevant features. With the relevance-based K-partition, we are now able to define a seed feature that can provide information about multivariate feature-to-feature relationships.

#### **Definition 10**.

Seed Feature. A feature *f*_*s*_ is a seed feature if ∀*f*_*j*_∈*F*∖*Ω*_*K*_,*f*_*sj*_ are (*ε*-)KJ-strongly relevant feature-pairs, where distinct features *f*_*j*_ are all coupled to the same feature *f*_*s*_.

For any strongly relevant feature-pair, if the coupled feature is identical, then the other features are dependent on the seed feature and might have interdependence among them to some extent in terms of biological interaction. Consequently, seed feature sets are defined for constructing putative Markov blankets.

#### **Definition 11**.

Seed Feature Set.

For a given *ε*>0, we consider *all* feature-pairs *f*_*sj*_ with seed feature *f*_*s*_ that are *ε*-KJ-strongly relevant according to Definition 6. A seed feature set, *M*_*s*_, is then a set of features led by *f*_*s*_ that has an underlying order of features w.r.t. their R1-Correlation:

(19) $$ M_{s}=\left\{\,f_{sj}\right\}=\left\{\,f_{s}:f_{j}|R\left(\,f_{j},C\right) \ge R\left(\,f_{j+1},C\right)\right\}.  $$

Here, ‘ *f*_*s*_:*f*_*j*_’ denotes that the first element in *M*_*s*_ is *f*_*s*_ followed by its coupled features *f*_*j*_.

Thus, a seed feature set consists of features based on (*ε*-)KJ-strongly relevant feature-pairs that have the same seed (leading) feature *f*_*s*_. We note that when the seed feature set is formed, feature-pairs *f*_*sj*_ are decoupled; that is, *M*_*s*_ is the collection of single features with an underlying order according to *R*(*f*_*j*_,*C*). Furthermore, *R*(*f*_*s*_,*C*)≥*R*(*f*_*j*_,*C*) is not necessarily true, but, by definition, it is part of all *ε*-KJ-strongly relevant feature pairs *f*_*sj*_. Thus, no matter how strong/weak *R*(*f*_*s*_,*C*) is, *f*_*s*_ is still considered as the first element in *M*_*s*_. Hence, *M*_*s*_ is called a set of features led by *f*_*s*_. We emphasise that Definition 11 uses the R2-correlation for strongly relevant feature-pairs as in Definition 6 with the same coupled feature *f*_*s*_, but the R1-correlation determines the underlying order within *M*_*s*_. The set allows us to look at feature-feature relationships beyond low-order interaction, which leads to the notion of redundant features with respect to a seed feature set.

#### **Definition 12**.

Redundant Feature. A feature *f*_*i*_∈*M*_*s*_ is redundant iff *f*_*i*_ is irrelevant with respect to {*M*_*s*_∖ *f*_*i*_}, i.e.,

(20) $$ p(C|\,f_{i},M_{s}\backslash\, f_{i})=p(C|M_{s}\backslash\, f_{i}).   $$

Although it seems that every feature within a set led by a seed feature is of relevance (strong or weak), in fact some features may not increase the predictive power with respect to the set. These features are then redundant and should be removed from the set. Therefore, given a seed feature set, we need another measure (i.e. R4-Correlation) to assess feature redundancy w.r.t. *M*_*s*_. In the present framework, we use conditional mutual information to calculate how strongly a feature variable is correlated with a class variable conditioned on *M*_*s*_ so that the redundant features can be identified, according to the following corollary.

#### **Corollary1**.

Criteria for Redundancy.

*f*_*i*_∈*M*_*s*_ is redundant iff

(21) $$ \text{CMI}(f_{i},C|M_{s}\backslash f_{i})=0.   $$

#### *Proof*.

Conditional mutual information can be expressed as the Kullback-Leibler Divergence (*D*_KL_), i.e., CMI (*X,Y*|*Z*)=*D*_*KL*_(*p*(*X,Y*|*Z*)∥*p*(*X*|*Z*)*p*(*Y*|*Z*))≥0. CMI (*X,Y*|*Z*) is equal to zero iff *p*(*x,y*|*z*)=*p*(*x*|*z*)*p*(*y*|*z*) for some assignment of values *x,y, z*. Since *p*(*x,y*|*z*)=*p*(*x*|*z*)*p*(*y*|*x,z*), we have *p*(*y*|*x,z*)=*p*(*y*|*z*), which implies *p*(*C*| *f*_*i*_,*M*_*s*_∖ *f*_*i*_)=*p*(*C*|*M*_*s*_∖ *f*_*i*_). In terms of Eq. (20), this means that *f*_*i*_ is redundant. □

After the removal of redundant features related to the seed feature set, one can eventually build a parsimonious set of features.

#### **Definition 13**.

Parsimony Model. ${M_{s}^{p}}$ is called a parsimony model iff ∀*f*_*i*_∈*M*_*s*_,*f*_*i*_ is not redundant within *M*_*s*_, which implies ${M_{s}^{p}}=M_{s}$.

A parsimony model is, therefore, a heuristic approximation of the Markov blanket where the existence of least feature redundancy is admitted. Initially, strongly relevant feature-pairs with the same seed feature are discovered for a putative blanket *M*_*s*_ in a forward phase. Out of these coupled features, some features could become false positives from a multivariate point of view. An approximate Markov blanket can then be created if these false positives are identified and eliminated from *M*_*s*_. Once multiple parsimony models are built, a set of candidate genes for high-throughput gene expression profiles can be selected.

#### **Definition 14**.

Candidate Genes.

A set *G* of features with $G \subseteq \cup _{u} {M_{s}^{p}}[\!u]$ is called a set of candidate genes such that $\forall {M_{s}^{p}}[\!u]$ & ${M_{s}^{p}}[\!u+1], R\left (\,{f_{s}^{u}},C\right)>R\left (\,f_{s}^{u+1},C\right)$.

As our original intention is to select gene-expression candidate genes from gene synergy, the parsimony model would not always be the best way to find a suitable size of a gene signature that not only would have good predictive power but also could reveal highly likely regulators or markers regarding a certain disease.

### The new filter

A complete framework for finding high-throughput gene-expression candidate genes is presented through Algorithm ??. The filter is named iRDA, an abbreviation for gene selection derived from **i**nterdependence with **R**edundant-**D**ependent analysis and **A**ggregation scheme. The framework is based on information-theoretic measures, heuristic search strategies, parameter estimation criteria, a mixture of forward-backward phases, and a gene aggregation scheme. The rationale for devising such a framework is to select a set of candidate genes from gene synergy that could potentially discover genetic regulatory modules or disease-related factors. Interdependence between features is, therefore, a matter of concern.



The proposed gene selection method is a four-step framework with a vast body of feature-pairs, including a set of analyses of feature relevance, feature interdependence, feature redundancy and dependence, and feature aggregation. The construction of the relevance-based K-partition is the main objective in the first step. The discovery of {*Ω*_*k*_} plays an important role in exploring strongly relevant feature-pairs, finding a parsimony model, and performing gene aggregation. In our framework, symmetrical uncertainty is used as R-Correlation in order to quantify the strength of association between features/feature-pairs and class variables. First of all, for each feature *f*_*i*_,SU_*i,c*_ (see Eq. 6) is calculated for estimating the degree of feature relevance (line A1:1). This is followed by sorting all of the calculated correlations in descending order (line A1:2); k-mean clustering is executed on the sorted list of SU_*i,c*_ in order to partition features into five groups that are labelled as *Ω*_1_,⋯,*Ω*_5_ in descending order according to their centroids of SU_*i,c*_ values (line A1:3). These feature types will be passed onto subsequent steps of the framework as indicators for the discovery of seed features, putative parsimony models, and a set of candidate genes.

The consideration of high-order gene interactions could have the potential for a road map of feature interdependence. However, because of the immense complexity of gene regulatory mechanisms, it would not be a good strategy to infer high-order feature interdependence in a direct way, since it is impractical to perform exhaustive search for visiting all feature-pairs if the number of features is very large. In large-scale HTS gene expression profiling, differentially expressed genes are biologically assumed to be a small portion of the population and the correlation values between a feature variable and a class variable are exponentially distributed. By using the K-partition {*Ω*_*k*_}, we are able to explore potential KJ-strongly relevant feature-pairs whose R2-Correlation values are beyond a threshold *ε*, which is estimated by the following method (see Definition 6).

#### **Definition 15**.

Criteria for *ε* Estimation.(a) A feature pair *f*_*i*_,*f*_*j*_∈{*Ω*_*k*_}, where *f*_*i*_ is ahead of *f*_*j*_ in {*Ω*_*k*_}, is called positive joint feature-pair iff

(22) $$ R\left(\,f_{ij},C\right)>R\left(\,f_{i},C\right); R\left(\,f_{j},C\right) > R\left(\,f_{i},f_{j}\right).  $$

(b) For given *L*(=100) positive joint feature-pairs, *ε* is defined by the mean of their R2-Correlation:

(23) $$ \varepsilon=\frac{\sum_{l=1}^{L} R_{l}\left(\,f_{ij},C\right)}{L}.  $$

Condition (a) implies that the feature-pair has a joint effect relative to a class variable that is more significant than the contribution of each single feature, where the contribution is still larger than the correlation between the two features.

In the second step, given a joint random variable of two features *f*_*i*_ and *f*_*j*_ (or *f*_*ij*_), joint symmetrical uncertainty SU_*i,j*;*c*_ is used to measure the strength of correlation between a feature-pair and a class variable. The key idea of interdependence is to generate seed feature sets by using forward selection (see line A1:4–5).

In the forward phase (see Algorithm 2), *Ω*_5_ is assumed to be a KJ-irrelevant-feature subset, while features with KJ-strong/weak relevance would exist in the other subsets of the partition. Moreover, if we assume that the population of *Ω*_1_ consists of predominantly strongly relevant features with a minority of weakly relevant features, then one feature from *Ω*_1_ in conjunction with other features from *Ω*_1_,…,*Ω*_4_ might constitute feature-pairs (line A2:1–12) whose joint symmetrical uncertainty values are greater than the threshold *ε* (line A2:3). A feature-pair *f*_*ij*_ having a strong R2-Correlation according to Definition 6 is added to a subset led by a seed feature *f*_*i*_ and/or to a subset led by a seed feature *f*_*j*_. Thus, *f*_*ij*_ can lead to two seed feature sets, *M*_*i*_ and *M*_*j*_, respectively. Due to the structure of the relevance-based K-partition, *R*(*f*_*i*_,*C*) is stronger than *R*(*f*_*j*_,*C*). Features with the strongest R1-Correlation, e.g., *f*_1_ and the respective pairs *f*_1*j*_, might generate a large number of *M*_*j*_, each consisting only of a few elements, which is too complex to be analysed. Moreover, such “fragmented seed sets” might generate noisy data and make gene aggregation extremely demanding. Consequently, we propose three selection modes (Greedy, Semi-Greedy, and Non-Greedy) for the production of probable seed feature sets. In the “Greedy” strategy, *f*_*ij*_ will be added to *M*_*i*_ only, and *f*_*i*_ is followed by *f*_*j*_. Since *f*_*i*_ is a seed feature, it will be added just once (line A2:4–6). On the other hand, *f*_*ij*_ is added to both seed feature sets *M*_*i*_ and *M*_*j*_ for the other two selection modes (line A2:7–9). In case of “Semi-Greedy” selection, we consider the removal of “fragmented seed sets” that have just two features, *f*_*s*_ and *f*_*i*_, inside. If *R*(*f*_*s*_,*C*) is weaker than *R*(*f*_*i*_,*C*), the fragment is removed; otherwise, the fragment would still be viewed as a candidate *M*_*s*_ (line A2:14–19). Eventually, a collection of non-empty seed feature sets, *G*_pre_, is returned (line A2:20). In summary, the “Greedy” strategy ignores many probable seed feature sets, but reduces the level of noise when genes are aggregated. The “Non-Greedy” selection is to fully explore the space of potential *M*_*s*_, and this is especially appropriate for a data matrix where only a few “fragments” are generated. The “Semi-Greedy” strategy not only allows the presence of some “fragments” (for not missing out on some true positives), but also takes targets false positives to be removed.



After a seed feature set has been formed, features are analysed with respect to redundancy in conjunction with a given seed features set. Thus, the third step in Algorithm 1 is to identify and remove redundant features with the aim of building a parsimonious set of features (see Definition 13). The analysis of redundancy and dependency will be carried out using backward elimination. Since there are three modes of selection in the forward phase, two different scenarios are considered in the backward procedure. Whereas the “Greedy” selection performs two runs of the backward phase with an insertion phase (line A1:7–10), the other two go only through the backward phase (line A1:11–12).

Algorithm 3 shows the details of the backward selection for generating a parsimony model ${M_{s}^{p}}$ (see Definition 13). Given a collection of subsets *G*_pre_, derived from interdependent analysis, the conditional mutual information CMI (*f*_*i*_,*C*|*M*_*s*_) (see Eq. 5) of a feature *f*_*i*_ and label *C* conditioned on a subset *M*_*s*_∈*G*_pre_ is chosen to be the R4-Correlation (see Definition 5(d)). The Corollary 1 reveals how to identify whether or not a feature is redundant with respect to a subst. However, it is inherent to HTS profiling that the data exhibit small sample sizes. Consequently, it is to be expected that the CMI-based correlation does not accurately express the exact joint distribution of features. Therefore, the redundant-dependent analysis of *M*_*s*_ will be base upon an approximation of backward elimination as defined below.

#### **Definition 16**.

Approximate Backward Elimination.We assume that elements of *M*_*s*_ are ordered in descending order according to the selected R1-Correlation (see Definition 11).

- (a) First Seat Last Check: ∀*f*_*i*_∈*M*_*s*_∖*f*_*s*_, the features are checked for redundancy in ascending order of R1-Correlation (least R1-value first) using the criteria of Corollary 1 and *f*_*s*_ is checked at last step;
- (b) Once *f*_*i*_ is removed, *f*_*i*_ cannot enter *M*_*s*_ again;
- (c) If *f*_*s*_ is removed, then *M*_*s*_ is discarded.

According to Definition 11 of a seed feature set, the front features in the seed feature set are of stronger relevance, which implies that they are less likely to be removed when Corollary 1 is applied. Therefore, following Definition 16, for any *M*_*s*_ ∈ *G*_pre_, we test if the value of CMI (*f*_*i*_,*C*|*M*_*s*_∖*f*_*i*_) is zero for every feature checked as described above (line A3:2–5). A feature whose CMI-value is zero will instantly be removed (line A3:3) and the next feature will be checked until all features from *M*_*s*_ have been tested. If a seed feature is eliminated, the subset *M*_*s*_ led by this feature will be discarded (line A3:4); otherwise, features that remain in the (potentially reduced) subset are considered to be dependent with regard to the seed feature. Thus, a subset that is not discarded is defined by at least two features (line A3:7).

In the forward phase with “Greedy” strategy, a potential feature-pair *f*_*sj*_ is not evenly included into seed feature sets. For this reason, we design an insertion phase for re-structuring putative seed feature sets, which is shown in Algorithm 4. For any $f_{\textit {sj}}\! \in \!G_{\text {pre}}^{\prime }$ generated by the first round of backward elimination, we add the pair to the seed feature set led by feature *f*_*j*_, if it is applicable. The move is motivated as follows: If the existence of *f*_*j*_ is in multiple seed feature sets after backward elimination, it might imply that *f*_*j*_ is likely to be a potential feature such that a seed feature set led by *M*_*j*_ might improve the overall performance.



Since the insertion phase might create new seed feature sets, a second round of the backward phase is executed (line A1:10). The complete execution of the third step of iRDA eventually returns a set *G*_post_ of multiple parsimony models ${M_{s}^{p}}$ (see Definition 13), where we assume that the elements (${M_{s}^{p}}$) of *G*_post_ are ordered, namely according to the R1-Correlation of their seed features that lead the parsimony models ${M_{s}^{p}}$ (line A1:13). The order of ${M_{s}^{p}}$ in *G*_post_ can be an indicator for gene aggregation (line A1:14–18).

### Wrapper-based evaluation scheme

The underlying paradigm of our method is to provide multiple parsimonious gene sets instead of a unique parsimony model as usually returned by existing feature selection methods. Such filters produce candidate genes sequentially one by one, which then extends also to the evaluation process. Unlike existing gene selectors, the iRDA method (see Algorithm 1) selects a candidate gene set *G* from *G*_post_, which is derived from parsimonious sets ${M_{s}^{p}}[\!u]$; i.e., iRDA aggregates candidate genes sequentially one set by one set, not one gene by one gene. Furthermore, the sets ${M_{s}^{p}}[\!u]$ are ranked according to the R1-correlation of their seed features $R({f_{s}^{u}},C)$ (see Definition 14).

Consequently, in order to cope with single gene vs parsimonious sets, it is imperative to provide the necessary implements for a fair comparison of candidate genes derived from different filters. We propose a wrapper-based evaluation scheme for evaluating different sets of candidate genes from various filters. Before presenting the proposed evaluation scheme, we introduce a set of three evaluation measures that are used to assess the classification performance of candidate genes.

#### **Definition 17**.

Performance Measures. Given a gene expression data set *D*, a set of candidate genes *G*, and the kNN classifier, the three performance measures of binary classification are denoted by

- (a) Error(*G*): generalization error;
- (b) AUC(*G*): area under the ROC curve;
- (c) MCC(*G*): Matthews correlation coefficient.

The generalization error (Error) is an intuitive judgment about the misclassification rate, but might not present a valid picture if the two classes under consideration strongly differ in size. The Matthews correlation coefficient (MCC) is generally viewed as a balanced summary statistics that takes into account true positives & negatives as well as false classifications [47]. The receiver operating characteristic (ROC) curve is a plot of the true positive rate (benefits) against the false positive rate (costs) for a given predictor [48]; while a random predictor leads to the AUC value of 0.5, the perfect outcome returns the AUC value of 1.

The proposed evaluator is based on a wrapper approach that utilises two performance measures (MCC and AUC) in conjunction with the k-Nearest Neighbours classification model (kNN). Additionally, a search scheme of sequential forward selection (SFS) is ‘wrapped around’ the application of MCC, AUC, and kNN. The evaluator is denoted by MA-kNN (MA is from the two measures MCC and AUC). If a set of candidate genes *G* is given, through the evaluation of genes one by one, conducted by a non-parametric classification model kNN, the behaviour of candidate genes can be evaluated by dual performance measures and based upon a sequential forward strategy. Here, AUC(*G*) and MCC(*G*) are chosen to find ‘promising’ genes, which are called successive victory genes, as defined below. This way an evaluation profile (Eval) of *G* is generated.

#### **Definition 18**.

Successive Victory Gene.Let *O* denote the set of previously examined genes; *u*(maxMCC) represents the uniqueness of maxMCC(*O* ∪ *f*). Then *g* is called a successive victory gene iff

(24) $$ g=\left\{ \begin{array}{ll} \text{arg}\left(\underset{\forall f}{\max}\text{MCC}(O\cup f)\right), & u(\max\text{MCC})=\text{TRUE},\\ \text{arg}\left(\underset{\forall h}{\max}\text{AUC}(O\cup h)\right), & u(\max\text{MCC})=\text{FALSE}.\\ \end{array}\right.  $$

The value of *u*(maxMCC) indicates how many of the genes examined along with *O* have an identical value maxMCC(*O* ∪ *f*). In case of *u*(maxMCC)=TRUE, there is a unique gene that dominates the performance measure MCC, and therefore gene *f* with maxMCC(*O* ∪ *f*) is selected. On the other hand, if *u*(maxMCC)=FALSE, then there are multiple genes along with *O* that have the same maximum of MCC, and therefore the additional measure AUC is invoked. Among the genes, a gene *h* with maxAUC(*O* ∪ *h*) is selected, i.e., the selected feature was successively ‘victorious’ in terms of MCC and AUC performance.

The MA-kNN evaluator, shown in Algorithm 5, begins with initial assignments of the examined gene set (*O*), the maximum of MCC measures (maxMCC), and the maximum of AUC values (maxAUC) (line A5:1). Since iRDA generates candidate genes from *G*_post_, the initial state of *O* is therefore the first parsimonious gene set, while for other filters the first gene identified by the filter is given to *O*. We then update *G* by removing the initial genes and construct an initial evaluation point *E*_*i*_ (line A5:2–4).



The two evaluation measures AUC(*O*∪*f*) and MCC(*O*∪*f*) are then computed for each gene *f* in *G*, and a successive victory gene *g* can be identified out of the remaining candidate genes by using sequential forward selection (line A5:7–19). The gene *g* is now removed from *G* and added to *O* (line A5:20–21), and the next evaluation point *E*_*i*_ is created for the update of *O* (line A5:22). This process is iteratively repeated until all genes in *G* have been examined, which means that an evaluation profile (Eval) of candidate genes has been obtained (line A5:24).

## Results and discussion

### Cancer benchmark datasets

Seven publicly available microarray-based gene expression benchmarks were used (see in Table 2, where IR is the imbalance ratio) to demonstrate that the proposed framework is potentially capable of selecting the most discriminative candidate genes for phenotype prediction and of finding significant genetic regulation within the selected set of genes. The seven datasets have frequently been used to validate the performance of cancer classification and gene selection (the data repositories are provided in Additional file 1: Table S1)

**Table 2:** Cancer-related gene expression profiling benchmarks

Dataset	Class	Samples	Genes	IR	Source
1. Brain	GBM/AO	50 (28/22)	12,625	1.27	(Nutt et al., 2003 [54])
2. CNS	Survivor/failure	60 (21/39)	7129	1.86	(Pomeroy et al., 2002 [55])
3. Colon	Negative/positive	62 (40/22)	2000	1.82	(Alon et al., 1999 [49])
4. Leukemia	ALL/AML	72 (47/25)	7129	1.88	(Golub et al., 1999 [56])
5. Lung	MPM/ADCA	181 (31/150)	12,533	4.84	(Gordon et al., 2002 [57])
6. Lymphoma	DLBCL/FL	77 (58/19)	7129	3.05	(Shipp et al., 2002 [58])
7. Prostate	Tumor/normal	102 (52/50)	12,600	1.04	(Singh et al., 2002 [59])

The Brain experiment was designed to investigate whether high-throughput gene expression profiling could classify high grade gliomas better than histological classification. This data set consists of 50 samples and 12,625 probe-sets using Affymetrix Human Genome U95Av2 Array. Out of 50 high grade gliomas, there are 28 glioblastomas (GBM) and 22 anaplastic oligodendrogliomas (AO). The second experiment recorded embryonal tumor patients in the central nervous system (CNS). There are 60 patient samples with 7129 genes. Among these samples, 21 are survivors (patients who are alive after treatment) while 39 are failures (patients who succumbed to their disease). The Colon experiment, introduced by Alon [49], consists of 62 samples from the patients of colorectal cancer, where 22 normal labels are extracted from healthy tissues and 40 abnormal biopsies are extracted from colon tumors. Out of more than 6500 genes in the original design of experiment, 2000 genes were selected to analyze by [49], based on the confidence at the measured expression levels. The Leukemia dataset includes gene expression profiles of two classes of bone marrow samples labeled with acute lymphoblastic leukemia (ALL) and acute myeloid leukemia (AML). There are 72 samples (47 ALL and 25 AML) and 7129 genes in this dataset. The fifth experiment is about clinically relevant cancer diagnostic tests of the Lung. There are 181 tissue samples profiled by 12,533 gene expression intensities. Among these observations, 31 are of malignant pleural mesothelioma (MPM) and 150 are of adenocarcinoma (ADCD). The Lymphoma experiment was designed to delineate diffuse large B-cell lymphoma (DLBCL) from a related germinal center B-cell lymphoma, follicular lymphoma (FL), and to identify rational targets for intervention. In this dataset, there are 77 observations (58 DLBCL and 19 FL) with the interrogation of 7129 probe-sets. The last dataset contains the expression levels of 12,600 genes for correlates of clinical Prostate cancer behavior. There are 102 observations in total, from 52 tumor patients (labeled as tumor) and 50 non-tumor patients (labeled as normal), respectively.

### Classification performance

To evaluate the effectiveness and characteristics of the proposed framework, three well-known multivariate filters (mRMR, CMIM, and FCBF) that utilise information theoretic measures are used for comparison through the examination of the seven recent microarray-based cancer classification datasets. Since the sample size is far smaller than the feature dimension in a typical HTS gene expression experiment, the conventional training-test data partition of 70–30 % (also known as holdout validation) is not very appropriate for the evaluation of gene selection approaches. Thus, the procedure of leave-one-out cross-validation (LOOCV) is used in our experiments. We employ performance measures for assessing the gene discrimination of the gene selectors under consideration. The three performance measures were introduced in Definition 17 (Error, AUC, and MCC), along with the kNN reference classifier. The classifier is used to induct candidate genes identified by a filter-based feature selector into a learning process. Here, we exploit a non-parametric classifier, kNN (for k = 3), for building inductive models from the results produced by gene selectors.

#### Parsimony model

Based upon parsimonious models (see Definition 13), minimal feature subsets are returned by the iRDA filter and the three reference filters are evaluated by using the three evaluation measures Error, AUC, and MCC. In particular, we proceed as follows: The maximum cardinality of ${M_{s}^{p}}$ (max$|{M_{s}^{p}}|$) that constitute *G*_post_ is identified. Each reference filter produces genes one by one, and every round the newly produced gene along with previously examined genes are evaluated. The evaluation process stops when max$|{M_{s}^{p}}|$ genes have been generated; if max$|{M_{s}^{p}}| <5$, the process stops when five genes have been evaluated. For each reference filter and each performance measure (Error, AUC, and MCC), a minimal gene set with the best performance is reported as a parsimony model for the filter and performance measure. Out of the ${M_{s}^{p}}$ constituting *G*_post_, iRDA reports the parsimony model with the best performance regarding the same evaluation measures, and the outcome is then compared to the parsimony models returned by reference filters.

In the respective tables, the best performance (within a row) is highlighted in **boldface**, and the second best evaluation result is highlighted in *italics*. Table 3 shows the generalization errors of mRMR, CMIM, FCBF, and iRDA over the seven microarray-based benchmarks. In six out of seven cases, iRDA returns the smallest number of misclassification, with three rate values identical to mRMR. For iRDA, the average error rate is 3.97 %, which is achieved for the smallest average number of 2.85 genes compared to the other three filters. On the third dataset, iRDA takes the second place. Although FCBF uses only a slightly larger average number of 3 genes, its discrimination levels are not as good as iRDA on all seven datasets. On average, mRMR is ranked at second place with an average rate of 5.3 %, and the average number of genes (3.71) is a bit smaller than that of CMIM (3.86). Both iRDA and mRMR have no misclassification on the Leukemia data, using three and four genes, respectively. All the parsimonious gene sets of four filters in terms of generalisation error rate are provided in Additional file 2: Table S2.

**Table 3:** Generalisation error rate of parsimony models over seven benchmark data using four information theoretic filters

	mRMR		CMIM		FCBF		iRDA	
	%	#	%	#	%	#	%	#
Brain	*6*	4	8	5	**4**	4	**4**	3
CNS	*18.33*	2	*18.33*	2	16.67	6	**13.33**	3
Colon	**6.45**	5	9.68	2	9.68	2	*8.06*	2
Leukemia	**0**	4	*1.39*	5	*1.39*	3	**0**	3
Lung	*1.1*	2	**0.55**	5	**0.55**	4	**0.55**	3
Lymphoma	**1.3**	4	*2.6*	2	24.68	1	**1.3**	3
Prostate	**3.92**	5	*4.9*	6	*4.9*	5	**3.92**	3
avg	5.3	3.71	6.49	3.86	8.84	3.57	**4.45**	**2.85**

%: misclassification rate; #: number of explored genes

The results of the AUC performance are summarised in Table 4. While iRDA displays the best results on five datasets, mRMR returns the same value (100 %) on Leukemia and is better on the Lymphoma datasets. In particular, the parsimony model of iRDA achieves 100 % on the Leukemia (also mRMR and CMIM) and Lung datasets. On the Prostate dataset FCBF has the best AUC performance with 97.67 % while iRDA and CMIM perform almost equally well (96.77 % vs 96.83 %). Overall, iRDA exhibits on average the highest AUC (98.11 %) with the fewest genes (4.14). The average AUC of CMIM and mRMR are 97.15 % and 96.23 %, respectively. The parsimonious gene sets of four filters in terms of AUC performance are provided in Additional file 3: Table S3.

**Table 4:** Area under the ROC curve of parsimony models over seven benchmark data using four information theoretic filters

	mRMR		CMIM		FCBF		iRDA	
	%	#	%	#	%	#	%	#
Brain	97.4	5	*98.86*	4	94.72	5	**99.68**	3
CNS	87.18	4	89.68	4	*91.15*	5	**94.08**	5
Colon	92.5	4	*96.25*	5	89.72	3	**98.86**	4
Leukemia	**100**	4	**100**	4	*99.96*	3	**100**	3
Lung	*99.94*	4	99.66	5	97.08	4	**100**	4
Lymphoma	**99**	5	*98.77*	4	50.36	5	97.37	3
Prostate	*97.62*	7	96.83	6	**97.67**	7	96.77	7
avg	96.23	4.71	97.15	4.57	88.67	4.57	**98.11**	**4.14**

%: AUC performance rate; #: number of explored genes

The results for the Matthews correlation coefficient are shown in Table 5. While mRMR returns the best results on four instances, iRDA achieves the best result on CNS and Leukemia data, with the second best performance on the remaining five instances. However, except for the Colon dataset, the difference between the first place and iRDA on four datasets (Brain, Lung, Lymphoma, and Prostate) is relatively small, with a maximum of 0.15 %. Moreover, with respect to the average value over all seven datasets, iRDA shows the best MCC performance with 91.89 %, which is achieved with the smallest average number of genes. We note that the ranking of filters w.r.t. average performance is similar to the one from Table 3, which is in line with the general observation that both generalization error and Matthews correlation coefficient can exhibit the same overall predictive power. The parsimonious gene sets of four filters in terms of MCC performance are provided in Additional file 4: Table S4

**Table 5:** Mathew correlation coefficient of parsimony models over seven benchmark data using four information theoretic filters

	mRMR		CMIM		FCBF		iRDA	
	%	#	%	#	%	#	%	#
Brain	87.96	4	84.61	5	**92.26**	4	*92.11*	3
CNS	*68.64*	2	*68.64*	2	67.08	6	**71.51**	3
Colon	**85.54**	5	78.63	2	78.63	2	*83.35*	4
Leukemia	**100**	4	96.95	5	*97.01*	3	**100**	3
Lung	96.11	2	**98.1**	5	*98.05*	4	*98.05*	3
Lymphoma	**96.62**	4	93.01	2	0.18	5	*96.5*	3
Prostate	**92.23**	5	90.65	6	90.36	5	*92.15*	3
avg	89.62	3.71	87.23	3.86	74.80	4.14	**90.52**	**3.14**

%: MCC performance rate; #: number of explored genes

#### Gene aggregation evaluation

Other than the construction of parsimonious subsets of genes, it is also important to identify candidate genes that could have a high classification performance and therefore are likely to play a role in regulatory modules or as biomarkers. As already mentioned, existing filters produce candidate genes sequentially one by one, and then this sequential order of genes is used to look at their classification performance. In contrast, iRDA is a filter that produces candidate genes by sequentially aggregating parsimonious gene sets. In this section, we use the sequential ordering of aggregated parsimonious gene sets and compare the classification performance to the three reference filters. For each dataset, we aggregate all of the parsimonious sets in *G*_post_, where the individual sets are dissolved and feature pairs are decoupled, with the resulting set being *G* (see aggregation part in Algorithm ??). With the known cardinality of *G*, each reference filter then produces the same number of genes in a sequence. We note that FCBF cannot generate as many genes as |*G*| for the CNS and Colon datasets.

**Fig. 2:**
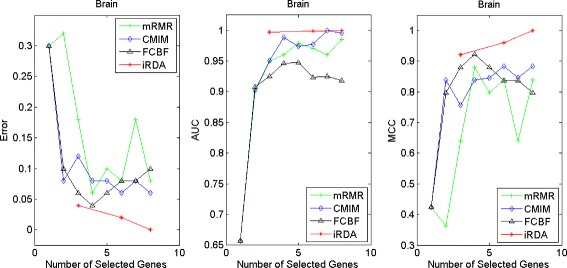
Classification performance of candidate genes found by four filters upon three measures: *brain* cancer

**Fig. 3:**
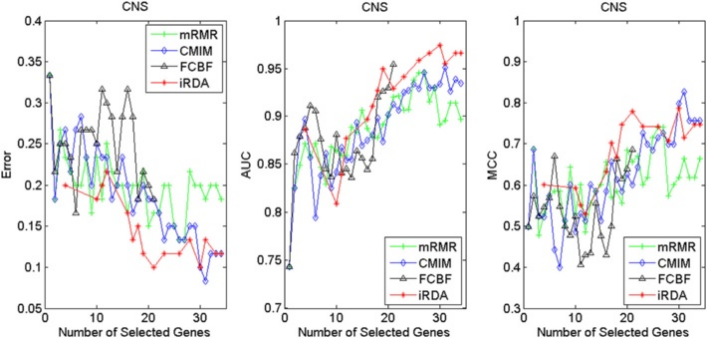
Classification performance of candidate genes found by four filters upon three measures: *CNS*

**Fig. 4:**
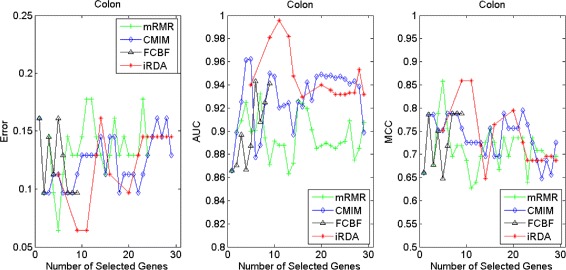
Classification performance of candidate genes found by four filters upon three measures: *colon* cancer

**Fig. 5:**
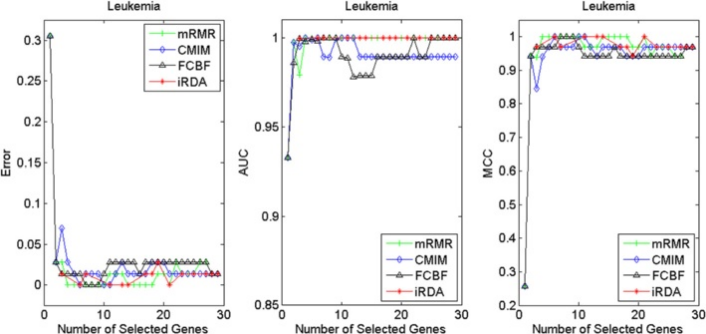
Classification performance of candidate genes found by four filters upon three measures: *leukemia* cancer

**Fig. 6:**
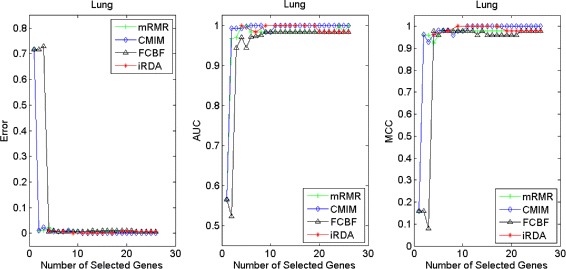
Classification performance of candidate genes found by four filters upon three measures: *lung* cancer

**Fig. 7:**
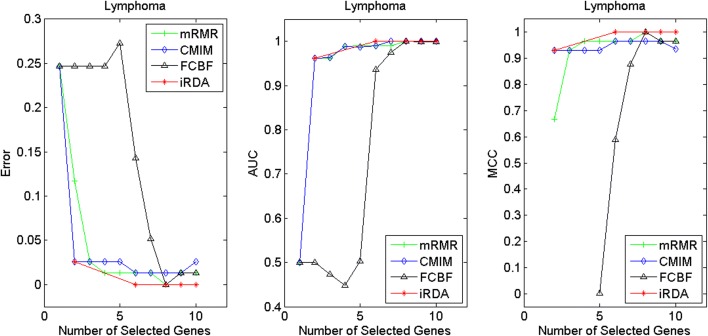
Classification performance of candidate genes found by four filters upon three measures: *lymphoma* cancer

**Fig. 8:**
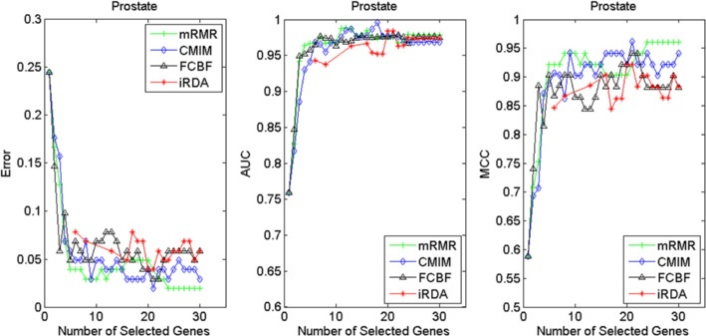
Classification performance of candidate genes found by four filters upon three measures: *prostate* cancer

Figures 2, 3, 4, 5, 6, 7 and 8 display the classification performance of the candidate genes produced by four filters across the seven microarray-based gene expression profiles with regard to three performance measures. For the Brain, CNS, and Colon data, iRDA produces the best discriminating genes, dominating all three performance measures, followed by CMIM and FCBF, while the genes selected by mRMR have a lower level of discrimination. We note that except for iRDA, no other filter dominates the three measures of Error, AUC, and MCC. Furthermore, while iRDA achieves for individual numbers of genes and the three measures Error, AUC, and MCC the levels of 0, 100, 100 %, respectively, on Leukemia, Lung, and Lymphoma data, genes produced by CMIM and mRMR reach the perfect level only on Leukemia and Lung data. FCBF exhibits a slightly worse performance in these datasets. However, since there is only a marginal difference between the filters for all three measures on Leukemia, Lung, and Lymphoma data, the three datasets are apparently more easily to classify w.r.t. the underlying two tissue types. For the Prostate dataset, mRMR has the best performance for the Error and MCC measures, whereas CMIM approaches the best level for AUC. Initially, iRDA has the worst performance on Prostate data (along with FCBF), but with an increasing number of genes its AUC performance improves and approaches the levels of mRMR and CMIM.

In summary, except for the Prostate dataset, iRDA dominates the performance results for an increasing number of genes. On the other hand, the parsimonious gene sets of mRMR can sometimes dominate top-rankings in MCC performance, as discussed in Section “Parsimony model”, but it seems that its discriminative power does not improve when more genes are selected. Furthermore, on the datasets we analysed, the performance of CMIM improves with an increasing number of genes, which in most cases eventually leads to better results than those produced by mRMR and FCBF.

#### Evaluation by MA-kNN wrapper

In addition to the performance analysis executed in Section “Gene aggregation evaluation” directly for the three measures Error, AUC, and MCC, we expose the gene sets produced by the four filters to the wrapper-based MA-kNN evaluator introduced in Section “Wrapper-based evaluation scheme”. Here, we are using the same candidate gene sets as described at the beginning of Section “Gene aggregation evaluation”, but the candidate genes are processed by the MA-kNN wrapper, and the outcome is then evaluated by the three performance measures as in Section “Parsimony model” (Tables 3, 4 and 5) and Section “Gene aggregation evaluation” (Figs. 2, 3, 4, 5, 6, 7 and 8).

**Fig. 9:**
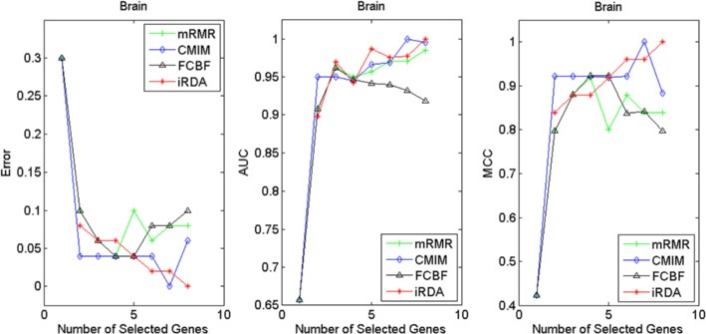
Processing of candidate genes by MA-kNN wrapper and evaluation by Error, AUC, MCC: *brain* cancer

**Fig. 10:**
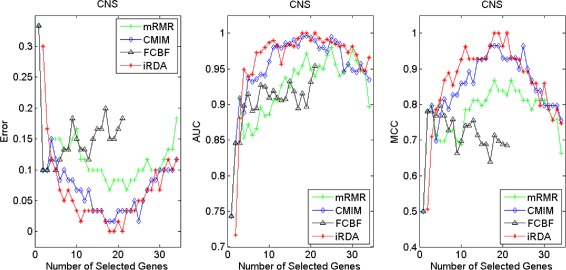
Processing of candidate genes by MA-kNN wrapper and evaluation by Error, AUC, MCC: *CNS*

**Fig. 11:**
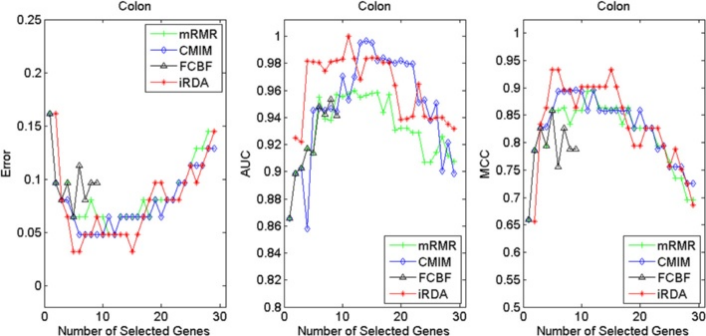
Processing of candidate genes by MA-kNN wrapper and evaluation by Error, AUC, MCC: *colon* cancer

**Fig. 12:**
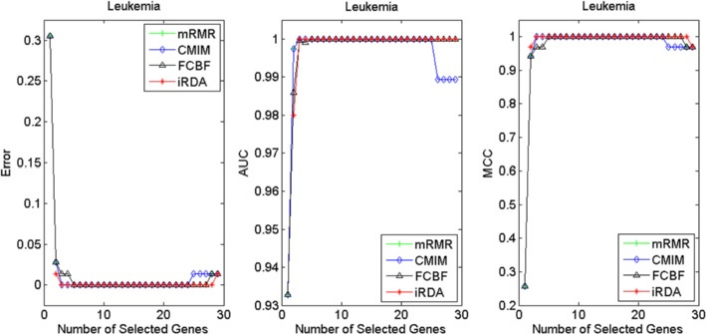
Processing of candidate genes by MA-kNN wrapper and evaluation by Error, AUC, MCC: *leukemia* cancer

**Fig. 13:**
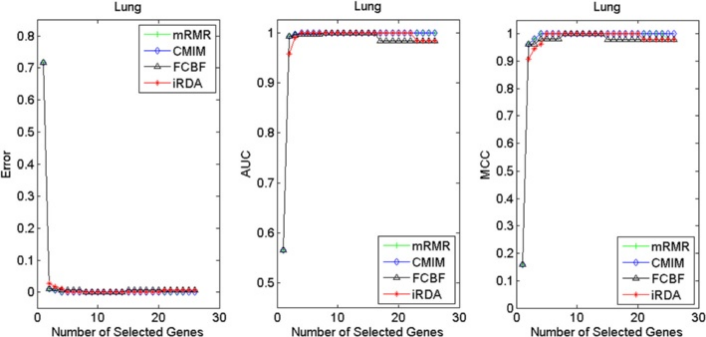
Processing of candidate genes by MA-kNN wrapper and evaluation by Error, AUC, MCC: *lung* cancer

**Fig. 14:**
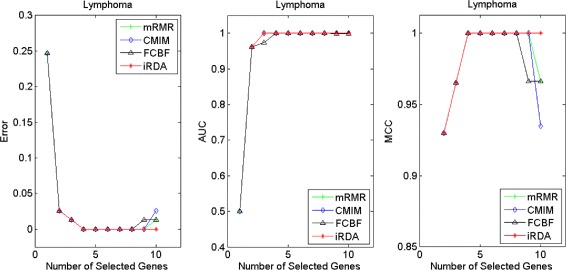
Processing of candidate genes by MA-kNN wrapper and evaluation by Error, AUC, MCC: *lymphoma* cancer

**Fig. 15:**
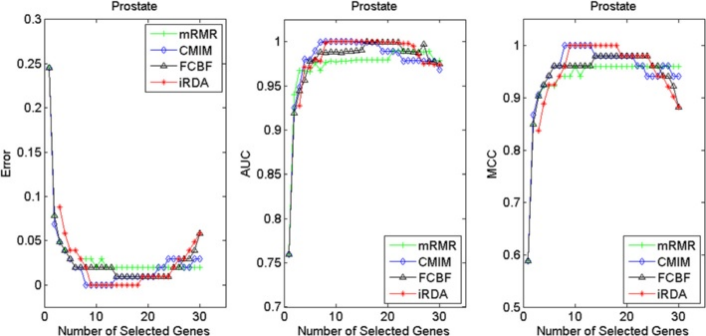
Processing of candidate genes by MA-kNN wrapper and evaluation by Error, AUC, MCC: *prostate* cancer

The results are shown in Figs. 9, 10, 11, 12, 13, 14 and 15. On the datasets for Brain, CNS, and Colon, the four methods demonstrate markedly different capacities of discrimination between binary samples. The new filter iRDA exhibits on the three datasets overall the best classification results and dominates for most of the gene numbers all of the three performance measures. The second best overall performance is displayed by CMIM, which for some gene numbers returns better results than iRDA. CMIM is followed by mRMR and FCBF, although FCBF shows sometimes marginally better results for Error and MCC than mRMR on Brain data for an increasing number of genes. We note that for CNS and Colon data, only iRDA achieves the optimum values of 0 and 100 % for AUC and MCC measures, respectively. On Leukemia, Lung, and Lymphoma data, all of the four filters perform nearly equally and perfectly well. Regarding the Prostate instance, the differences of performance among the four methods become less obvious than those for the Brain, CNS, and Colon data. However, one can observe that overall iRDA dominates the the other three filters, followed by CMIM, which also reaches optimum levels for Error, AUC, and MCC. FCBF is ranked third and reaches the optimum level for AUC, while mRMR displays on Prostate data the least performance. In comparison to the results from Figs. 2, 3, 4, 5, 6, 7 and 8, iRDA demonstrates an even stronger overall performance when the MA-kNN wrapper is applied. All the candidate genes selected by the four filters are provided in Additional file 5: Table S5.

### Other disease types

Besides the cancer expression profiling benchmarks, we have carried out the gene expression experiments on additional disease types in order to further understand the characteristics of the new filter. The experiments concern a variety of diseases and include larger samples, all above 100 and up to 173, and their detailed information is provided in Table 6. All the datasets are archived in Gene Expression Omnibus (GEO) [50] and can be accessed by their GSE Accession Number. The series GSE755 is to profile multiple myeloma (MM) patients with (Y) and without (N) bone lytic lesions by MRI. GSE8759 is to study cultured skin fibroblasts from Marfan syndrome (MS) subjects and unaffected controls of similar age and sex distributions. The identification of gene expression level in different tissues between HIV-positive and HIV-negative patients is represented by GSE30310. The experimental design of the last dataset is about the Alzheimer’s like neurodegeneration (AD), using the anti-NGF AD11 transgenic mouse model, which is compared to transgenic VH controls. Table 7 shows the classification performance of the parsimonious models of genes selected by the four filters over the diseases multiple myeloma, Marfan syndrome, HIV infection, and neurodegeneration. CMIM dominates the first place in the average of generalisation error rates (8.21 %), while mRMR and iRDA have the best performance regarding the average of AUC scores (95.36 %) and MCC scores (79.64 %), respectively. In terms of the minimal subset of selected genes, the three filters perform not significantly differentially and outperform FCBF.

**Table 6:** Additional disease gene expression profiling experiments

Disease	Class	Samples	Genes	IR	Source	GEO
A. Multiple Myeloma (MM)	Lytic lesion: N/Y	173 (36/137)	12,625	3.81	(Tian et al., 2003 [60])	GSE755
B. Marfan Syndrome (MS)	Control/case	101 (41/60)	4132	1.46	(Yao et al., 2007 [61])	GSE8759
C. HIV Infection (HIV)	Negative/positive	166 (41/125)	4776	3.05	(Morse et al., 2012 [62])	GSE30310
D. Neurodegeneration (AD)	VH/AD11	119 (59/60)	16,515	1.02	(D’Onofrio et al., 2011 [63])	GSE63617

**Table 7:** Classification performance of parsimony models over four disease gene-expression data using four information theoretic filters

	mRMR		CMIM		FCBF		iRDA	
Error	%	#	%	#	%	#	%	#
Multiple Myeloma (MM)	**14.45**	7	**14.45**	6	15.03	10	16.18	10
Marfan Syndrome (MS)	**3.96**	4	6.93	7	11.88	3	*5.94*	6
HIV Infection (HIV)	13.86	8	11.45	8	15.06	7	**10.84**	8
Neurodegeneration (AD)	0.84	2	**0**	2	0.84	2	**0**	2
avg	8.28	5.25	**8.21**	5.75	10.7	5.5	*8.24*	6.5
AUC	%	#	%	#	%	#	%	#
Multiple Myeloma (MM)	90.71	9	90.09	9	**91.26**	10	89.84	9
Marfan Syndrome (MS)	**98.84**	4	97.2	3	93.25	7	*98.76*	4
HIV Infection (HIV)	91.88	8	92.42	8	87.29	7	**92.49**	8
Neurodegeneration (AD)	**100**	2	**100**	2	**100**	2	**100**	2
avg	**95.36**	5.75	94.93	5.5	92.95	6.5	*95.27*	5.75
MCC	%	#	%	#	%	#	%	#
Multiple Myeloma (MM)	61.52	7	**63.67**	6	61.55	10	59.56	10
Marfan Syndrome (MS)	**91.93**	4	85.7	7	78.67	6	*88.61*	6
HIV Infection (HIV)	63.06	8	68.98	8	57.99	7	**70.4**	8
Neurodegeneration (AD)	98.33	2	**100**	2	98.33	2	**100**	2
avg	78.71	5.25	79.59	5.75	74.14	6.25	**79.64**	6.5

%: performance rate; #: number of explored genes

**Fig. 16:**
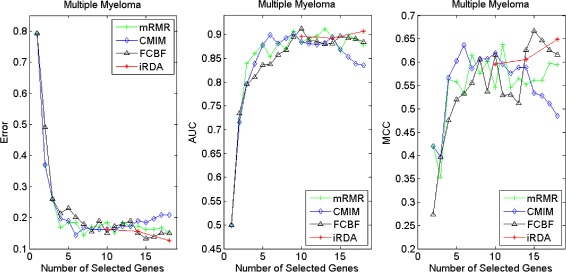
Classification performance of candidate genes found by four filters upon three measures: *Multiple Myeloma*

**Fig. 17:**
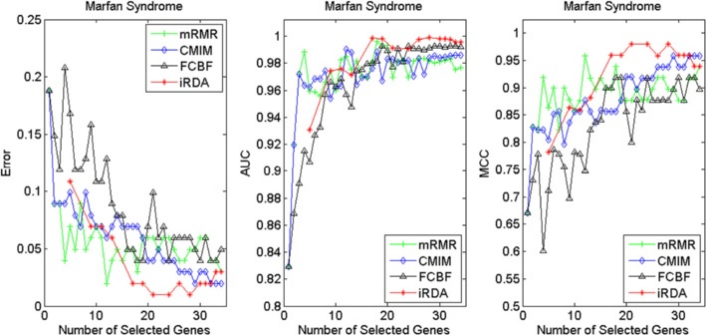
Classification performance of candidate genes found by four filters upon three measures: *Marfan Syndrome*

**Fig. 18:**
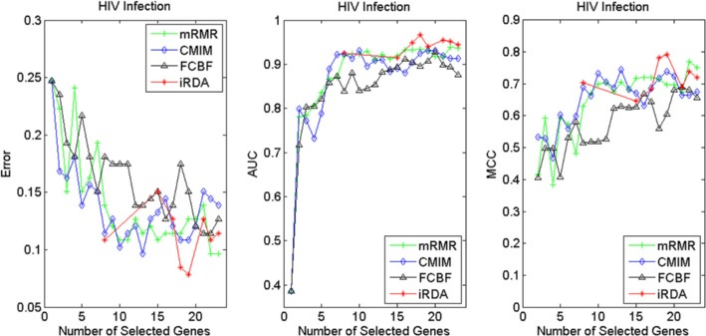
Classification performance of candidate genes found by four filters upon three measures: *HIV Infection*

**Fig. 19:**
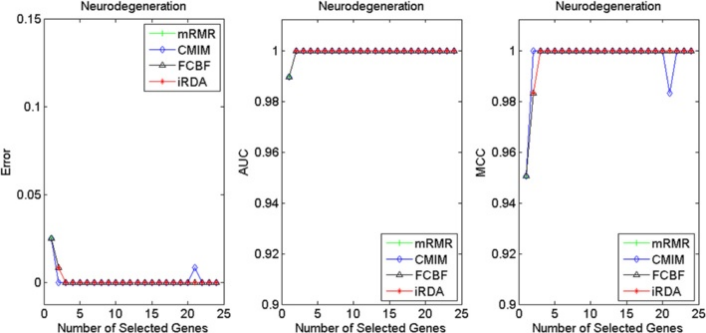
Classification performance of candidate genes found by four filters upon three measures: *Neurodegeneration*

**Fig. 20:**
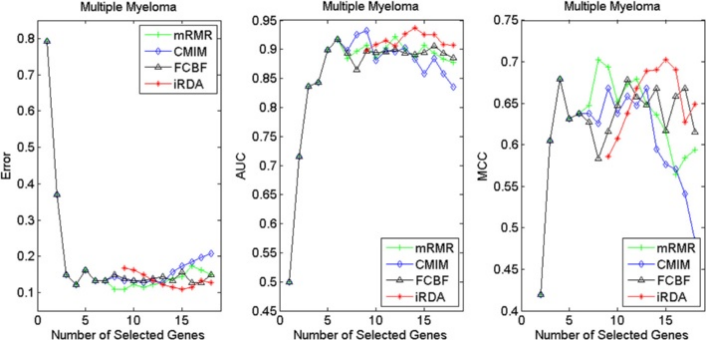
Processing of candidate genes by MA-kNN wrapper and evaluation by Error, AUC, MCC: *Multiple Myeloma*

**Fig. 21:**
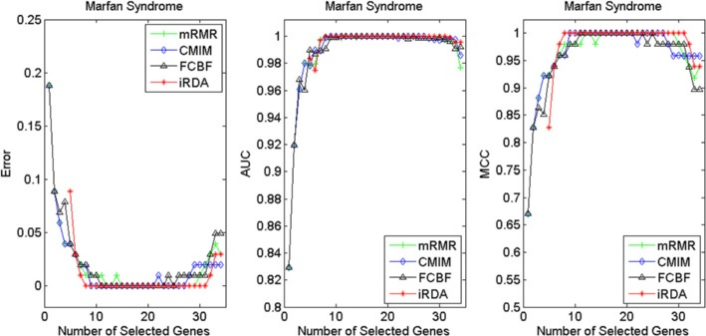
Processing of candidate genes by MA-kNN wrapper and evaluation by Error, AUC, MCC: *Marfan Syndrome*

**Fig. 22:**
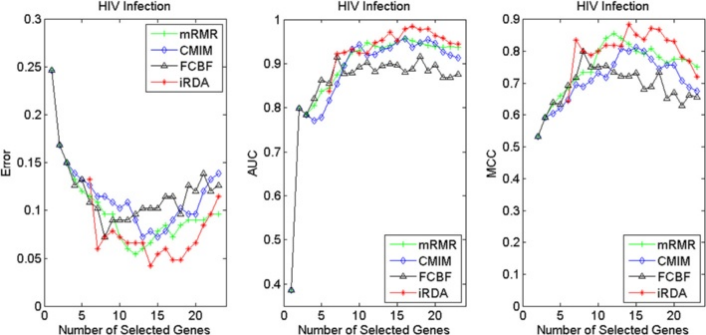
Processing of candidate genes by MA-kNN wrapper and evaluation by Error, AUC, MCC: *HIV Infection*

**Fig. 23:**
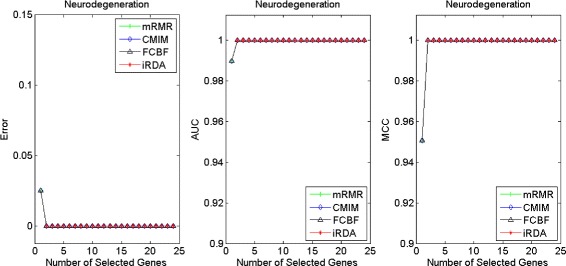
Processing of candidate genes by MA-kNN wrapper and evaluation by Error, AUC, MCC: *Neurodegeneration*

Figures 16, 17, 18 and 19 display empirical results about whether or not the performance can be improved if more genes are selected. In the experiments for MS and HIV, iRDA outperforms the other three methods and achieves the evaluation values of 0, 100, and 100 % for Error, AUC and MCC; whereas CMIM returns values similar to mRMR, and both are better than FCBF. For the AD dataset, all filters perform equally, no matter what measures are used. For the disease of multiple myeloma, CMIM performs worst, and there is no strong distinction between iRDA, FCBF and mRMR regarding the measures Error and AUC. Finally, the classification performance of selected genes that are evaluated by using the MA-kNN wrapper for the four datasets is shown in Figs. 20, 21, 22 and 23. The experimental results show that iRDA outperforms the other methods on all four diseases, while mRMR takes the second place, followed by FCBF and CMIM. We also observe that the AD experiment is data set that can be easily classified, and that MM is the most difficult to classify, which can be seen from the MCC performance with a level of around 0.7. All the candidate genes selected by the four filters are provided in Additional file 5: Table S5.

### Computation time

We conducted an experiment about computation time with regard to the four gene selectors applied to all of the 11 gene expression profiling data in the environment of MATLAB 7.14, with the hardware being an Intel Core i7-3820 CPU of 3.60 GHz and a 24 GB RAM. For each of the four filters, we measured the time required for generating candidate genes. The results are summarised in Table 8. Not surprisingly, improved performance comes at a price: CMIM is the fastest method, followed by mRMR, FCBF, and iRDA. Since CMIM and mRMR are criteria-based only filters and do not incorporate search-based methods, they are expected to be faster than FCBF and iRDA. Although both FCBF and iRDA utilise a heuristic search strategy, there are two selection phases (forward and backward) involved in iRDA. Consequently, FCBF outperforms iRDA in terms of run-time on 7 out of the 11 data sets considered in our study. Moreover, we note that iRDA generates more viable parsimonious sets to build candidate genes, which, of course, affects the run-time. However, all the iRDA run-time data are in the region of a few seconds.

**Table 8:** Computation time for gene selectors

	mRMR	CMIM	FCBF	iRDA
Brain	0.21	0.03	1.69	5.61
CNS	0.53	0.02	0.78	3.15
Colon	0.13	0.01	0.17	0.08
Leukemia	0.51	0.05	0.97	3.42
Lung	1.27	0.10	4.16	2.92
Lymphoma	0.17	0.02	0.96	3.12
Prostate	1.06	0.06	1.68	8.18
Multiple Myeloma	0.88	0.05	2.10	7.92
Marfan Syndrome	0.39	0.03	0.49	0.59
HIV Infection	0.41	0.03	0.59	0.43
Neurodegeneration	1.35	0.07	2.52	1.13
avg	0.63	0.04	1.46	3.32

Unit: Seconds

### Stability performance

In order to assess the robustness of the four feature selection methods, we consider two stability index-based measures with respect to differently sized gene lists. The Jaccard index quantifies the amount of overlap between two datasets, ranging from 0 to 1, with 0 indicating empty intersection and 1 indicating that the two sets are equal (see also Tanimoto distance [51]).

#### **Definition 19**.

Jaccard Index. Given two gene lists *G*_*i*_,*G*_*j*_,JI(*G*_*i*_,*G*_*j*_) a stability index called Jaccard index, which is defined as follows:

$$\text{JI}(G_{i},G_{j})=\frac{|G_{i} \cap G_{j}|}{|G_{i} \cup G_{j}|}. $$

The definition is extended to larger sets of gene lists in the following way:

#### **Definition 20**.

Overall Jaccard Stability. Given a system of *l* gene lists *U*, ∀*G*_*i*_,∀*G*_*j*_∈*U* we define the overall Jaccard stability of *U* as

$$S_{\text{JI}}(U)=\frac{2}{l(l-1)} \sum_{i=1}^{l-1} \sum_{j=i+1}^{l} \text{JI}(G_{i},G_{j}). $$

The Jaccard index suffers from the problem of list-size-bias: The more lists approach the size of the total pool of features, the higher the probability of an overlap in pairs of gene lists. To solve the problem, the relative weighted consistency [52] has been introduced based on the relative degree of randomness of the system of lists in the feature selection process.

#### **Definition 21**.

Relative Weighted Consistency. Given a system *U* of *l* gene lists *G*_*j*_⊆*F*, let *o*_*j*_(*f*_*i*_)=1 denote *f*_*i*_ ∈ *G*_*j*_ (zero, otherwise). We set $N = \sum _{j=1}^{l} \sum _{i} o_{j}(\,f_{i})$, which is the total number of occurrences of features in *U*, and $R_{f} = \sum _{j=1}^{l}o_{j}(\,f)$. The relative weighted consistency of *U* is then defined by

$$\begin{array}{@{}rcl@{}}  & & S_{\text{RWC}}(U,F) \\  & = &\frac{|F|(N\,-\,Q\,+\,\sum_{f \in F} R_{f}(R_{f}\,-\,1))\,-\,N^{2}\,+\,Q^{2}}{|F|(q^{2}\,+\,l(N\,-\,q)\,-\,Q)\,-\,N^{2}\,+\,Q^{2}}, \end{array} $$

where *Q*=*N*(mod |*F*|) and *q*=*N*(mod *l*).

**Fig. 24:**
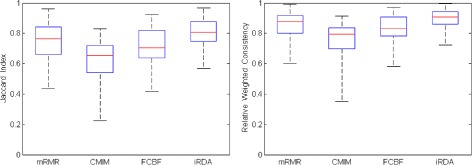
Box plots of the Jaccard index and the Relative Weighted Consistency (RWC) to show the stability of the four filters

We compare the stability of the four filters by using the two stability measures *S*_JI_ and *S*_RWC_ over the eleven datasets (seven cancer benchmarks and four disease experiments). For each dataset, a pool of data samples *S*_*j*_ derived from the procedure of leave-one-out (LOO) is constructed. For example, the Brain dataset consists of 22+28=50 samples, which generates 50 sets *S*_*j*_ of size 49. For each *S*_*j*_, a set of candidate genes ${G_{j}^{h}}$ is produced by each of the feature selection filters, *h*=1,…,4. For identifying the ${G_{j}^{h}}$, as before (see Sections “Gene aggregation evaluation” and “Evaluation by MA-kNN wrapper”), iRDA is executed first, which determines the cardinality of gene lists. The other three methods subsequently generate the same size of gene sets. Again, due to the nature of FCBF, the method can sometimes return only a smaller portion of genes. Figure 24 displays the results for the Overall Jaccard Stability and the Relative Weighted Consistency as average values over the eleven datasets. Although *S*_RWC_ provides higher values than *S*_JI_ does, the two box-plots show similar results. For both measures, iRDA is the most stable with the least variance, followed by mRMR with a smaller median value and a larger variance. FCBF is slightly inferior to mRMR, while the least stable selector is CMIM in both plots.

**Fig. 25:**
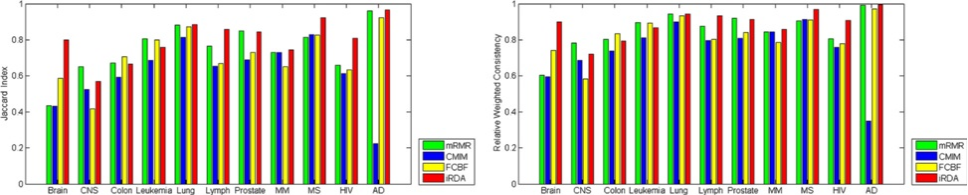
Bar charts of the stability of the four filters across eleven datasets using Jaccard index and RWC, respectively

The details of stability measure results on each dataset are shown in bar-charts in Fig. 25. The behaviour of the four filters is less stable on the Brain, CNS, Colon, and HIV datasets, except for iRDA on Brain and HIV data, when compared to the other seven datasets. The gene lists returned by iRDA perform better than by the other three filters on seven datasets, specifically on Brain data. The results for Lung data suggest that the gene lists are least varied, such that all methods perform nearly equally well with high index values. For the instances where mRMR dominates the other filters, the difference between iRDA and mRMR is only marginal. We noticed that there is a large amount of FCBF lists whose sizes are rather small on CNS and Colon data compared to the other three filters, but, interestingly, this causes FCBF to be the least stable on CNS data and the most stable on Colon data. Surprisingly, CMIM appears highly unstable on AD data, whereas the other three filters remain very stable.

### Enrichment analysis

Whilst a set of genes is selected, it is essential to understand if some genes would interact with other genes in the set. Gene set enrichment analysis (GSEA) is able to provide a good insight into the complex interaction among genes, based on collections of *a priori* biologically defined and annotated gene sets [53]. Since its introduction about 10 years ago, GSEA has become a standard procedure for looking at groups of genes that share common biological function, chromosomal location, or regulation. In the present paper, we utilised for the analysis of candidate gene sets the Molecular Signatures Database (MSigDB-v4) in conjunction with GSEA-v2 in order to gain knowledge about how many gene sets are statistically significantly enriched. Table 9 reports (i) the number of native features (denoted by N and the same as in Sections “Gene aggregation evaluation” and “Evaluation by MA-kNN wrapper”) produced by the four filters that were considered for the enrichment analysis over Brain, CNS, Leukemia, Lung, Lymphoma, and Prostate datasets, and (ii) the number of genes (denoted by C) that were actually used by GSEA after the process of collapsing original features into gene symbols. We excluded the Colon dataset, since it exploited an array of Affymetrix Hum6000 where many ESTs are mapped into the same gene symbol. All the collapsed genes over six cancer benchmarks for GSEA are provided in Additional file 6: Table S6.

**Table 9:** Features selected by the four filters and used in GSEA version 2 over various cancer-related gene expression experiments

	Brain		CNS		Leukemia		Lung		Lymphoma		Prostate	
	#N	#C	#N	#C	#N	#C	#N	#C	#N	#C	#N	#C
mRMR	8	8	34	31	29	28	26	25	10	10	30	30
CMIM	8	7	34	31	29	28	26	26	10	10	30	30
FCBF	8	8	21	20	29	28	26	25	10	10	30	29
iRDA	8	7	34	31	29	29	26	23	10	9	30	26

N: Native Features; C: Collapsed Features

Based on the sets of collapsed features, numbers of gene sets recognised by GSEA-v2 as statistically significant enrichment for the six cancer types are shown in Table 10, where GSEA employs a false discovery rate to indicate a significance level (FDR <0.25). The results show that iRDA produces on five out of the six datasets the largest number of statistically enriched gene sets. CMIM occupies the second place in this experimental study, whereas mRMR and FCBF exhibit a similar enrichment performance. Of particular interest is that, although, for iRDA fewer candidate genes are collapsed into gene symbols, the collapsed genes still produced a larger number of enrichment groups. For example, iRDA has 6, 19, and 16 enrichment groups based on 7, 9, and 26 collapsed genes for the Brain, Lymphoma, and Prostate instances, respectively, while there are a fewer enrichment groups identified for the other three gene selectors based upon a larger number of collapsed genes (see Table 9). We note that the Lung dataset is the most imbalanced (IR = 4.84) and that the number of samples (= 181, see Table 2) is also relatively larger compared to the other datasets. We found that iRDA does not perform well on the Lung dataset when compared to CMIM, although both filters display an almost identical classification performance on this particular dataset. From Table 10 we see that there is a far greater amount of enrichment groups for Leukemia data, independently of the underlying gene selector: GSEA returned 45, 21, 12, and 7 statistically significantly enriched gene sets for iRDA, CMIM, mRMR, and FCBF. The details of all the enrichment groups and genes are provided in Additional file 7: Table S7.

**Table 10:** Statistically significantly enriched gene sets of the four gene selectors analysed by using GSEA version 2

	Brain	CNS	Leuk.	Lung	Lymph.	Prost.
mRMR	1	0	12	1	1	0
CMIM	1	4	21	14	4	0
FCBF	0	0	7	0	3	0
iRDA	6	6	45	0	19	16

*Leuk.* Leukemia, *Lymph.* Lymphoma, *Prost.* Prostate

FDR <0.25

## Conclusions

A new filter, iRDA, for identifying gene-expression candidate genes for phenotype prediction derived from high-throughput screening technologies is fully introduced in this paper. The filter is able to produce small sets of discriminative genes, either in form of a parsimony model or as a set of candidate genes, with an impact on better phenotype prediction. The output produced by iRDA meets the demands of a domain user, since a small number of candidate genes is the preferred basis to perform *in vitro* validation efficiently.

The effectiveness of iRDA was validated on eleven datasets, including seven well-known cancer benchmarks and four additional disease experiments. Based on the transcriptomic profiling data, iRDA was compared to the three information theoretic filters (mRMR, CMIM, and FCBF) in terms of classification performance, stability indices, and the gene set enrichment analysis (GSEA). According to the experimental results, we conclude that (1) Parsimonious sets generated by iRDA have good and comparable classification performance; (2) Candidate genes explored by iRDA dominate the sets produced by mRMR, CMIM, and FCBF; (3) iRDA exhibits on average the best stability with the smallest variance; (4) There are more sets of statistically significant enrichment in genes selected by iRDA than in those discovered by mRMR, CMIM, and FCBF. The performance results come at a price in terms of run-time. However, the gene selection is executed on all data sets within a few a seconds on standard desktop equipment. Overall, we think that the new iRDA filter has the potential of identifying genes that might have an inferior relevance, but contribute strongly to interactions between genes. Such genes, accompanied by other genes in a signature set, could have a measurable impact on phenotype distinction, which would not necessarily be seen at the level of expression data.

## Additional files

Additional file 1
**Table S1.** Data repositories. The file provides data repositories of seven cancer benchmarks summarised in Table 2. (XLSX 10 kb)

Additional file 2
**Table S2.** Parsimonious gene sets of error performance. The file provides all the gene sets of four filters over eleven datasets based on generalisation error rate from Tables 3 and 7. (XLSX 19 kb)

Additional file 3
**Table S3.** Parsimonious gene sets of AUC performance. The file provides all the gene sets of four filters over eleven datasets based on AUC measurement from Tables 4 and 7. (XLSX 20 kb)

Additional file 4
**Table S4.** Parsimonious gene sets of MCC performance. The file provides all the gene sets of four filters over eleven datasets based on MCC measurement from Tables 5 and 7. (XLSX 19 kb)

Additional file 5
**Table S5.** Candidate genes. The file provides all the candidate genes selected by four filters over eleven datasets to be evaluated in Figs. 2, 3, 4, 5, 6, 7, 8, 9, 10, 11, 12, 13, 14, 15, 16, 17, 18, 19, 20, 21, 22 and 23. (XLSX 36 kb)

Additional file 6
**Table S6.** Collapsed Genes. The file is a supplement to Table 9 to provide all the collapsed genes over six cancer benchmarks for GSEA. (XLSX 15 kb)

Additional file 7
**Table S7.** GSEA Results. The file provides the details of all the enrichment groups and genes summarised in Table 10. (XLSX 29 kb)

## Abbreviations

HTS: High-throughput screening
mRMR: Minimum-Redundancy and Maximum-Relevance framework
CMIM: Conditional Mutual Information Maximization
FCBF: Fast Correlation-Based Filter
iRDA: Interdependence with Redundant-Dependent analysis and Aggregation scheme
AUC: Area under the receiver operating characteristic curve
MCC: Matthews correlation coefficient
kNN: The k-Nearest Neighbours classification model
MA-kNN: A wrapper based on MCC and AUC in conjunction with the kNN classifier
LOOCV: Leave-one-out cross-validation
IR: Imbalance ratio
GBM: Glioblastomas
AO: Anaplastic oligodendrogliomas
CNS: Central nervous system
ALL: Acute lymphoblastic leukemia
AML: Acute myeloid leukemia
MPM: Malignant pleural mesothelioma
ADCD: Adenocarcinoma
DLBCL: Diffuse large B-cell lymphoma
FL: Follicular lymphoma
MM: Multiple Myeloma
MS: Marfan Syndrome
HIV: HIV Infection
AD: Neurodegeneration
GSO: Gene Expression Omnibus
RWC: Relative Weighted Consistency
FDR: False discovery rate
GSEA: Gene set enrichment analysis
